# Complicated Grief Following the Traumatic Loss of a Child: A Systematic Review

**DOI:** 10.1177/00302228231170417

**Published:** 2023-05-11

**Authors:** Monique Jayde Champion, Meegan Kilcullen

**Affiliations:** 1 College of Healthcare Sciences, 104401 James Cook University, Townsville, QLD Australia

**Keywords:** bereaved parents, complicated grief, grief, coping/adaptation, attachment

## Abstract

Complicated grief is a disabling condition that occurs when the natural grief process is disturbed or prolonged. Research demonstrates that complicated grief is more prevalent following the sudden or violent loss of a child. Despite the high incidence of accidental death worldwide, little research has focused on parental grief trajectories following this form of traumatic loss. A systematic review was conducted to explore parental bereavement outcomes following accidental death. Studies were included if they specifically examined complicated grief in parents bereaved by the accidental death of their child. A total of 767 articles were identified and seven studies met the eligibility criteria for review. Poorer outcomes were identified in relation to the mode of death, relationship type, time post-loss, perceived support, perceived justice and comorbidities. Results of the current study may be used to inform the development of clinical practice guidelines for the treatment of complicated grief.

## Background

Bereavement, or the loss of a loved one through death, can be a life-changing experience (Dutta et al., 2019; Morris et al., 2019; Neimeyer, 2000a). Loved ones often contribute to an individual’s identity and sense of belonging, which gives life meaning and purpose (Keesee et al., 2008; Shear, 2015). Bereavement activates an instinctual grief response as those left behind attempt to find ways to live without their loved one (Enez, 2017; Shear, 2012; Shear et al., 2013; Tal Young et al., 2012).

Grief is a culture-bound experience that affects multiple domains of functioning (Enez, 2017; Mason et al., 2020; Tofthagen et al., 2017). It is associated with an acute period of intense suffering, characterised by extreme sorrow and yearning, that tends to abate over time (Fujisawa et al., 2010; Lundorff et al., 2017; Shear, 2012). Grief typically becomes integrated into ongoing life as time passes, such that the bereaved individual is able to accept the reality of the loss and slowly adjust to their new world (Bogensperger & Lueger-Schuster, 2014; Neimeyer, 2000a; Shear et al., 2017).

Complicated grief is the syndrome that emerges when the natural grief process is disturbed or prolonged (Field & Filanosky, 2010; Prigerson et al., 2009; Shear et al., 2013). It refers to a pervasive and disabling grief response that persists for a period beyond that which is considered adaptive (Lobb et al., 2010; World Health Organisation [WHO], 2019). Complicated grief is a serious health concern for a significant minority of bereaved individuals, with a well-established connection between this type of grief and excess morbidity and mortality (Fujisawa et al., 2010; Mason et al., 2020; Prigerson et al., 2009; Rogers et al., 2008; Wittouck et al., 2011). Complicated grief is more prevalent among those who have lost a child, particularly when the death is sudden or violent (Bekkering & Woodgate, 2021; Kersting et al., 2011; Morris et al., 2019; Shear, 2015; Wilson et al., 2022).

### Parental Bereavement

The loss of one’s child is recognised as one of the most painful and unnerving forms of bereavement (Lichtenthal et al., 2010; Reynolds et al., 2020; Rogers et al., 2008; Shear et al., 2013; Zetumer et al., 2015). The experience is considered inherently unnatural and out-of-order with the family life cycle, irrespective of the age of the child (Bekkering & Woodgate, 2021; Cohen-Mansfield et al., 2013; Keesee et al., 2008). Bereaved parents face a profound and enduring grief that challenges the concepts of healing and recovery (Dutta et al., 2019; Keesee et al., 2008; Lichtenthal et al., 2010; Rogers et al., 2008).

From an evolutionary perspective, grief is theorised to be a manifestation of attachment (Goldstein et al., 2020; Reynolds et al., 2020). Attachment refers to the innate behavioural system that motivates humans to maintain or regain proximity to significant figure(s) during stressful or threatening situations (Bowlby, 1980; Wijngaards-de Meij et al., 2007). It is deemed a reciprocal process designed to ensure survival of the young (Kearney & Byrne, 2015). The loss of one’s child provokes an overpowering sense of separation distress, leading bereaved parents to seek reunification with their missing loved one (O’Connor, 2012; Reynolds et al., 2020).

Accordingly, the natural grief process is thought to involve the modification of attachment from a physical connection to an emotional or spiritual one (Dutta et al., 2019; Rubin et al., 2017). Contemporary research indicates that the concept of continuing bonds with the deceased is a common and healthy response to bereavement (Jahn & Spencer-Thomas, 2018). As time passes, bereaved parents generally learn to “strike a balance between remembering the child they lost and moving forward with their lives” (Bekkering & Woodgate, 2021, p. 13). Bereaved parents are forced to recognise the loss as part of their new identity (Dutta et al., 2019).

### Traumatic Bereavement

A death that is sudden or violent compounds the distress associated with bereavement (Field & Filanosky, 2010). The cause of death may be unexplained or attributed to homicide, suicide or accident (Nakajima et al., 2012). These forms of traumatic loss often precipitate a crisis of meaning in which the predictability of the world is called into question (Keesee et al., 2008; Rogers et al., 2008). A lack of preparedness for the death, paired with a negative end-of-life experience, renders healing and recovery difficult (Jahn & Spencer-Thomas, 2018; Murphy et al., 2003).

To facilitate healing and recovery, meaning reconstruction is deemed the central task of grief (Neimeyer, 2000b). Humans develop orienting systems as a means of processing and understanding life events (Gerrish et al., 2009; Milman et al., 2019; Parkes, 2006). These orienting systems allow individuals to find meaning in life by providing an overarching sense of predictability and purpose (Gerrish et al., 2009; Keesee et al., 2008). Sudden or violent death tends to challenge such constructions of meaning, leaving bereaved individuals with minimal capacity to make sense of the loss or find any form of benefit (Bogensperger & Lueger-Schuster, 2014; Neimeyer, 2019). Those left behind are forced to rebuild or revise their assumptions of the world in order to accommodate the loss and construct a coherent life story (Calhoun et al., 2010; Milman et al., 2019).

### Rationale for Current Study

The sudden or violent loss of a child places bereaved parents at increased risk for developing complicated grief (Kersting et al., 2011; Morris et al., 2019; Shear, 2015; Wilson et al., 2022). Yet, the majority of bereavement research is centred on spousal loss with limited generalisability to other populations (e.g., Fujisawa et al., 2010; Mason et al., 2020). When other populations are considered, modes of death are frequently restricted to natural or expected causes, with sudden or violent death excluded from analysis as a confounding factor (e.g., Lundorff et al., 2017). Furthermore, research that is focused on traumatic parental bereavement often fails to differentiate between types of sudden or violent death despite potential variability in outcomes (e.g., Baddeley et al., 2015; Lichtenthal et al., 2013; Suttle et al., 2022).

Fatal injuries, encompassing most forms of sudden or violent loss, constitute a major global health concern and account for nearly 8% of all deaths across the world (WHO, 2021). The majority of these deaths are unintentional or accidental, caused by events such as road traffic injuries, falls, drowning, burns or poisoning (WHO, 2021). Road traffic injuries are in fact the leading cause of death among children and young adults aged 5–29 years worldwide (WHO, 2022). There is a clear need for research of bereavement resulting from these losses.

The aim of this systematic review was to identify research exploring complicated grief in parents bereaved by the accidental death of their child. It is important to consider the unique needs of bereaved parents in order to provide adequate support through their bereavement journey.

## Method

### Search Strategy

The present study was conducted in accordance with the Preferred Reporting Items for Systematic Reviews and Meta-Analyses (PRISMA) guidelines (Page et al., 2021). A comprehensive literature search was performed using the following electronic databases: PsycINFO (ProQuest), Medline, Emcare, CINAHL, Scopus, PubMed and Web of Science. The search string included both medical subject headings (MeSH terms) and free text (keywords) in an attempt to capture all relevant articles. The search string was informed by the Population, Intervention, Comparison, Outcome (PICO) framework (Sackett et al., 1997) and represented the three main concepts for review: parental bereavement, accidental death and complicated grief. The search terms are presented in Table 1. A manual search of references in all relevant articles was also undertaken. Searches were conducted in July 2021 and updated in November 2022.

**Table 1.** Search String.

Main Concept	Search Terms
Parental bereavement	(“Parent child relations” OR “mother child relations” OR “father child relations” OR parent* OR mother* OR father*) AND
Accidental death	((Accident* AND death) OR “accidental death” OR “violent death” OR “violent loss” OR “sudden death” OR “sudden loss” OR “child death” OR “child loss”) AND
Complicated grief	(“Complicated grief” OR “prolonged grief” OR “traumatic grief” OR “complex grief” OR “pathological grief”)

### Eligibility Criteria

Articles were selected for review based on the following inclusion criteria: (a) original empirical study, with quantitative and/or qualitative methodologies; (b) published in a peer-reviewed journal; (c) written in the English language; and (d) focused on complicated grief in parents bereaved by the accidental death of their child. The age of the child at the time of death was not restricted. Articles that primarily examined other modes of death unrelated to accidents were excluded (e.g., terminal illness, suicide, homicide, sudden infant death syndrome). Articles were limited to those published from 2000 to allow for a contemporary perspective.

### Data Extraction

A library of all identified articles was compiled using reference management software. After duplicates were removed, the first author independently conducted a two-step screening process. Titles and abstracts were initially screened for eligibility and articles that did not meet criteria were removed. All remaining articles were retrieved in full and assessed for final inclusion. The second author then screened all included studies, as well as those for which eligibility was uncertain. The first author independently extracted data from all included studies using a pre-defined data extraction template to summarise the key characteristics and outcomes.

### Quality Assessment

The Crowe Critical Appraisal Tool (CCAT) was used to assess the quality of all included studies (Crowe et al., 2012). Articles were examined in relation to eight categories: preliminaries, introduction, design, sampling, data collection, ethical matters, results and discussion. The CCAT is considered a valid and reliable tool for quality assessment (Crowe et al., 2012). Scores are allocated for each category using a 6-point Likert scale ranging from 0 to 5. Scores are then summed for a total maximum score of 40, with higher scores reflecting better quality research. The overall quality rating for each article is presented as a percentage.

## Results

**Figure 1.**
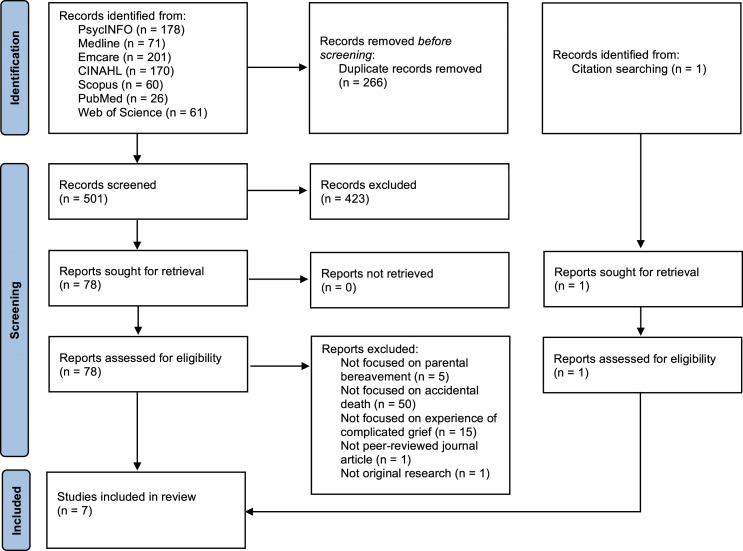
PRISMA flowchart of the study selection process.

A total of 767 articles were identified across seven databases (see Figure 1). Following the removal of 266 duplicates, the title and abstract of 501 articles were screened for eligibility and 423 articles were subsequently excluded. The remaining 78 articles were screened in full. Articles that did not focus on all three main concepts (parental bereavement, accidental death and complicated grief) were excluded. A stand-alone conference abstract was also excluded, as well as an article that did not present original research. The remaining six articles met the eligibility criteria for the current study. One further article was found following a manual search of references, bringing the total number of included studies to seven.

Table 2 presents a summary of the key characteristics and outcomes of all included studies, including overall quality ratings as per the CCAT. The studies were conducted in South Korea (Choi & Cho, 2020; Huh et al., 2017), Norway (Dyregrov et al., 2003; Kristensen et al., 2012), the United States (Feigelman et al., 2011), Cambodia (Field et al., 2014) and Belgium (Spooren et al., 2001). Five of the studies were cross-sectional in design (Choi & Cho, 2020; Dyregrov et al., 2003; Feigelman et al., 2011; Huh et al., 2017; Spooren et al., 2001). The remaining two studies utilised case-control (Field et al., 2014) and longitudinal (Kristensen et al., 2012) designs. Two of the studies incorporated mixed methods data (Dyregrov et al., 2003; Spooren et al., 2001), while all other studies contained quantitative research only (Choi & Cho, 2020; Feigelman et al., 2011; Field et al., 2014; Huh et al., 2017; Kristensen et al., 2012). Participants were predominantly mothers, with wide variability in time post-loss. Four of the studies involved mass disasters (Choi & Cho, 2020; Field et al., 2014; Huh et al., 2017; Kristensen et al., 2012) and the others were focused on drug-related accidents (Feigelman et al., 2011), traffic accidents (Spooren et al., 2001) or undifferentiated accidents (Dyregrov et al., 2003). All studies included a validated measure of complicated grief. Overall quality ratings differed considerably; however, all studies were deemed to be of acceptable quality. As methodological heterogeneity precluded a formal meta-analysis, a descriptive synthesis was undertaken to integrate the findings from each study.

**Table 2.** Summary of Included Studies.

Author (year)	Setting	Design	Sample	Study Population (% female; mean age; time post-loss)	Measures	Relevant Findings	Quality Rating
Choi & Cho (2020)	South Korea	Cross-sectional quantitative	Convenience sample of 122 parents bereaved by the Sewol ferry accident	58% female; 48.8 years; 2 years post-loss	Structured clinical interview for complicated grief (SCI-CG)	• 53.3% met criteria for both PTSD and complicated grief (PTSD/CG group); 37.4% met criteria for complicated grief only (CG only group); and 9.3% did not meet criteria for PTSD or complicated grief (resilient group)	75%
					Structured clinical interview for DSM-IV PTSD (SCID-PTSD)	• Compared to the resilient group, membership in the PTSD/CG group was predicted by lower perceived justice and female gender. No other significant differences were found	
					Perceived justice questionnaire (unvalidated)		
Dyregrov et al. (2003)	Norway	Cross-sectional mixed methods	Convenience sample of 232 parents bereaved by accidents (*n* = 68), suicide (*n* = 128) or SIDS (*n* = 36)	60% female; 40 years; 6–23 months post-loss	Inventory of complicated grief (ICG)	• For parents bereaved by accidents, 78% met criteria for complicated grief diagnosis; 51% experienced high levels of posttraumatic stress; and 72% scored above the cut-off score for high levels of psychosocial and physical complaints	73%
					Impact of events scale (IES-15)	• Parents bereaved by accidents reported significantly more problems across all scales than those bereaved by SIDS. There were no significant differences between scores for those bereaved by accidents versus suicide	
					General health questionnaire (GHQ-28)	• For parents bereaved by accidents, complicated grief was best predicted by social isolation and not having surviving children	
					Social interaction questionnaire (unvalidated)		
					Semi-structured interviews		
Feigelman et al. (2011)	United States	Cross-sectional quantitative	Convenience sample of 571 parents bereaved by drug-related accidents (*n* = 48), suicide (*n* = 462), natural causes (*n* = 24) or other accidents (*n* = 37)	Demographic information and time post-loss not reported	Inventory of complicated grief (ICG)	• There were no significant differences between scores for those bereaved by drug-related accidents versus suicide. No other groups were compared	55%
					Impact of events scale (IES-15)	• When comparing parents bereaved by drug-related accidents or suicide (combined) to those bereaved by natural causes or other accidents (combined), the former group reported significantly more problems across all scales except stigmatisation	
					Grief experience questionnaire (GEQ; unvalidated version)		
					Index of psychological problems		
					Depression scale (unvalidated)		
					Stigmatisation scale (unvalidated)		
Field et al. (2014)	Cambodia	Retrospective case-control quantitative	Purposive sample of 60 mothers bereaved by the Koh Pich bridge stampede with a matched control group of 59 non-bereaved women	100% female; 50.8 years; 6 months post-loss	Prolonged grief (PG-13)	• For bereaved mothers, 47.5% met criteria for complicated grief diagnosis; 57.5% met criteria for PTSD; 63.8% scored above the cut-off score for anxiety; and 61.3% scored above the cut-off score for depression	75%
					PTSD checklist (PCL-5)	• When comparing mean symptom levels for bereaved mothers versus the control group, bereaved mothers scored significantly higher across all scales. The incidence of disordered symptom levels (i.e., scores above the clinical cut-off) was significantly greater for bereaved mothers relative to the control group	
					Hopkin symptom checklist (HSCL-25) for anxiety and depression	• Group status (bereaved mothers vs. control group) was uniquely predicted by symptoms of complicated grief over-and-above all other symptom measures	
Huh et al. (2017)	South Korea	Cross-sectional quantitative	Convenience sample of 84 parents bereaved by the Sewol ferry accident	46.4% female; 47.4 years; 18 months post-loss	Inventory of complicated grief (ICG)	• 94% met criteria for complicated grief diagnosis; 70.2% reported clinically significant symptoms of posttraumatic stress; 65.5% reported clinically significant symptoms of posttraumatic embitterment; 50% reported severe levels of depression; 50% reported severe somatic symptoms; 11.9% reported severe insomnia; and 10% reported alcohol dependence	78%
					PTSD checklist (PCL-5)	• There were no significant differences between levels of psychological distress (complicated grief, PTSD or depression) for mothers versus fathers	
					Posttraumatic embitterment disorder (PTED) scale		
					Patient health questionnaire (PHQ-9) for depression		
					Patient health questionnaire (PHQ-15) for somatic symptoms		
					Insomnia severity index (ISI)		
					Alcohol use disorders identification test (AUDIT-C)		
Kristensen et al. (2012)	Norway	Prospective longitudinal quantitative	Convenience sample of 32 parents bereaved by military training accident	50% female; 51.8 years at time of accident; 1, 2, 5 and 23 years post-loss	Inventory of complicated grief (ICG)	• At the 23-year follow-up, there were no current cases of complicated grief; however, 60% reported one or more symptoms. The most commonly reported symptoms were yearning for the deceased, anger and bitterness	78%
					Mini-international neuropsychiatric interview (MINI)	• At the 23-year follow-up, only one of the 16 parents had a current psychiatric condition (anxiety disorder)	
					General health questionnaire (GHQ-20)	• When assessing longitudinal changes, symptoms of psychological distress and grief significantly reduced over time	
					Expanded Texas inventory of grief (ETIG)		
Spooren et al. (2001)	Belgium	Cross-sectional mixed methods	Convenience sample of 85 parents bereaved by traffic accidents	61.2% female; 48 years; mean 4 years post-loss	Inventory of complicated grief (ICG)	• 71.4% met criteria for complicated grief diagnosis and 72.6% experienced high levels of psychological distress	63%
					General health questionnaire (GHQ-28)	• The highest-rated symptoms of complicated grief were yearning for the deceased, difficulty accepting the death and anger. The lowest-rated symptom was avoidance of memories	
					Crisis support scale (CSS)	• Mothers scored significantly higher than fathers for symptoms of complicated grief	
					Service satisfaction questionnaire (unvalidated)	• Dissatisfaction with services at the time of the accident, specifically in terms of practical help and information about the circumstances of the death, was significantly associated with symptoms of complicated grief	
					Semi-structured interviews	• Poor perceived support, both at the time of the accident and at the time of the study, was significantly associated with symptoms of complicated grief	

Overall, researchers found increased levels of complicated grief in parents bereaved by the accidental death of their child. Prevalence rates varied from 47.5% following a crowd stampede in Cambodia (Field et al., 2014) to 94% following a ferry accident in South Korea (Huh et al., 2017). In a 23-year follow-up study of parents bereaved by a military training accident, Kristensen et al. (2012) found no current cases of complicated grief; however, 60% of bereaved parents still reported at least one current symptom. Poorer outcomes were identified in relation to the following themes—*Mode of Death; Relationship Type; Time Post-Loss; Perceived Support; Perceived Justice; and Comorbidities.*

### Mode of Death

Two of the included studies compared parental grief symptomology following accidental deaths versus other modes of death (Dyregrov et al., 2003; Feigelman et al., 2011). Dyregrov et al. (2003) compared three forms of traumatic loss: accidents, suicide and sudden infant death syndrome (SIDS). Complicated grief was measured using the Inventory of Complicated Grief (ICG), while posttraumatic stress was measured using the Impact of Events Scale (IES-15) and psychosocial distress using the General Health Questionnaire (GHQ-28). Parents bereaved by accidents reported significantly more symptoms across all scales than those bereaved by SIDS. Meanwhile, there were no significant differences between scores for parents bereaved by accidents versus suicide.

Feigelman et al. (2011) contrasted drug-related accidents with suicide, natural deaths and other accidents. The researchers hypothesised that parents bereaved by drug-related accidents would experience similar levels of grief difficulties, posttraumatic stress, psychological distress and stigmatisation to those bereaved by suicide. They also hypothesised that, when combined, parents whose children died from drug-related accidents or suicide would face poorer outcomes than parents whose children died from natural causes or other accidents. Indeed, there were no significant differences between scores for parents bereaved by drug-related accidents versus suicide. These parents experienced significantly more grief difficulties (including symptoms of complicated grief), posttraumatic stress and psychological distress when compared to those bereaved by natural deaths or other accidents. The experience of stigmatisation was not statistically different between groups. These findings are limited by the methodological decision to combine samples of parents bereaved by natural deaths or other accidents as a single comparison group. It is unlikely that these samples could be considered homogeneous.

### Relationship Type

Four of the included studies examined bereavement outcomes with respect to the type of relationship between the bereaved and the deceased (Choi & Cho, 2020; Dyregrov et al., 2003; Huh et al., 2017; Spooren et al., 2001). In Spooren et al.’s (2001) study of parents bereaved by traffic accidents, mothers experienced significantly greater levels of complicated grief than fathers. Choi and Cho (2020) identified similar findings in their study of the Sewol ferry disaster whereby being a mother significantly predicted the dual diagnosis of complicated grief and Posttraumatic Stress Disorder (PTSD). Interestingly, in an earlier study of the same ferry disaster, Huh et al. (2017) found no significant differences between mothers and fathers for symptoms of complicated grief or PTSD. Across both genders, Dyregrov et al. (2003) found that losing an only child was associated with poorer outcomes.

### Time Post-Loss

As most of the included studies were cross-sectional in design, changes over time could not be ascertained. Nevertheless, Kristensen et al. (2012) conducted a longitudinal study of parents bereaved following a military training accident. Data was collected at four time points (1, 2, 5 and 23 years post-loss) in relation to grief and psychological distress; however, complicated grief was only measured at the final follow-up. No parents met the diagnostic criteria for complicated grief 23 years post-loss. In general, the intensity of grief reactions and levels of psychological distress progressively declined over time.

### Perceived Support

Dyregrov et al. (2003) identified social isolation as the best predictor of complicated grief in parents bereaved by accidents. Half of all parents in this sample reported withdrawing from others following the loss of their child. Common reasons for social isolation were explored through semi-structured interviews. Parents described feelings of guilt and self-blame, as well as shifted values and priorities, as explanations for social withdrawal. Parents also referred to social isolation as a “response to the helplessness of social networks on how to encounter people in crisis” (p. 158). Parents suggested that it was important for their social networks to understand how to best support them in their grief.

Similarly, Spooren et al. (2001) investigated perceived support as a salient factor in bereavement outcomes. Results showed greater levels of complicated grief in parents who expressed dissatisfaction with support services at the time of the accident. Parents described a need for more practical help, as well as more detailed information about the circumstances of the death in order to “make a reconstruction of what happened” (p. 180). Results also showed greater levels of complicated grief in parents who expressed a lack of available support, either at the time of the accident or at the time of the study. Parents reported a significant decrease in perceived support over time.

### Perceived Justice

Two of the included studies explored the impact of perceived justice following a mass ferry disaster (Choi & Cho, 2020; Huh et al., 2017). Choi and Cho (2020) asked parents to rate their level of satisfaction in relation to five aspects of perceived justice: truth seeking, compensation, punishment, apology and the role of the government. Most parents (86.9%–95.1%) rated each item as “not at all satisfied”. Results demonstrated that lower perceived justice significantly predicted the dual diagnosis of complicated grief and PTSD. Correspondingly, Huh et al. (2017) measured perceived justice in relation to reactive embitterment—that is, a persistent negative emotion in response to injustice, insult or breach of trust. Most parents (65.5%) scored in the clinically significant range for symptoms of reactive embitterment at 18 months post-loss.

### Comorbidities

All except one (Kristensen et al., 2012) of the included studies referred to common comorbidities among parents with complicated grief. Across all studies, complicated grief most frequently co-occurred with PTSD (Choi & Cho, 2020; Dyregrov et al., 2003; Feigelman et al., 2011; Field et al., 2014; Huh et al., 2017). Parents with complicated grief also appeared to experience increased rates of depression (Feigelman et al., 2011; Field et al., 2014; Huh et al., 2017; Spooren et al., 2001), anxiety (Field et al., 2014; Spooren et al., 2001), somatic symptoms (Dyregrov et al., 2003; Huh et al., 2017; Spooren et al., 2001), insomnia (Huh et al., 2017; Spooren et al., 2001) and psychosocial complaints (Dyregrov et al., 2003; Spooren et al., 2001).

## Discussion

The intention of the current study was to identify research exploring complicated grief in parents whose children died in an accident. A total of seven studies met the eligibility criteria for review. The studies consistently demonstrated that parents bereaved by accidents are particularly at risk for poor adaptation to loss, with a much higher prevalence of complicated grief when compared to the general bereavement population (Choi & Cho, 2020; Dyregrov et al., 2003; Feigelman et al., 2011; Field et al., 2014; Huh et al., 2017; Kristensen et al., 2012; Spooren et al., 2001). While complicated grief has been reported to affect approximately 11% of all bereaved individuals (for a meta-analysis, see Lundorff et al., 2017), the included studies exploring parental bereavement following accidental death reported prevalence rates ranging from 47.5% (Field et al., 2014) to 94% (Huh et al., 2017). This finding highlights the unique needs of parents bereaved by the accidental death of their child.

It is well established that the aetiology of complicated grief is influenced by circumstances surrounding the bereavement (Fujisawa et al., 2010; Shear, 2012). In line with this, two of the included studies reported that the mode of death can impact parental grief symptomology (Dyregrov et al., 2003; Feigelman et al., 2011). Parents bereaved following accidents or suicide appeared to have comparable outcomes, while those bereaved following other forms of loss seemed to fare better. Feigelman et al. (2011) further suggested that different types of accidents, such as drug-related accidents versus traffic accidents, may lead to variable outcomes. It is possible that certain accidental deaths impose additional challenges for bereaved parents due to increased feelings of guilt, self-blame and/or shame (see Duncan & Cacciatore, 2015).

Many of the included studies referred to the nature of the relationship between the bereaved and the deceased (Choi & Cho, 2020; Dyregrov et al., 2003; Huh et al., 2017; Spooren et al., 2001). Mothers appeared to experience poorer outcomes than fathers, which is in accordance with previous literature that indicates women are especially vulnerable to complicated grief reactions (Fernandez-Alcantara & Zech, 2017; Jaaniste et al., 2017; Morris et al., 2019). Dyregrov et al. (2003) also identified that having surviving children served as a protective factor following parental bereavement. The impact of relationship type is likely related to expressions of attachment. The bond between mother and child reflects a prototypical relationship, uniquely strengthened through primary caregiving (Fernandez-Alcantara & Zech, 2017; Goldstein et al., 2020). The caregiving role is indeed integral to the identity of many bereaved parents (Boelen, 2016; Lichtenthal et al., 2010). The loss of a child, especially an only child, may be perceived as the loss of the caregiving identity. Those with remaining children perhaps endure forced re-engagement with life as necessitated by parenthood.

Kristensen et al. (2012) conducted the only longitudinal study included in this review. Unsurprisingly, symptoms of grief and psychological distress appeared to dissipate over time. As this was a 23-year follow-up study, it would be useful to determine at what point in time bereaved parents tend to shift from an acute (or complicated) grief reaction to a state of integrated grief. A better understanding of parental grief trajectories may allow for screening and monitoring of at-risk individuals within a preventative framework (Mason et al., 2020). It may also assist with the provision of more individualised support that complements the dynamic phases of healing and recovery (Morris et al., 2019).

Perceived support was identified as a key determinant of bereavement outcomes (Dyregrov et al., 2003; Spooren et al., 2001). Poor social support may occur as a consequence of social isolation, perhaps due to psychological distress. Conversely, the average social network may be ill equipped to provide adequate support to bereaved parents. Spooren et al. (2001) acknowledged that the delivery of appropriate support services at the time of the accident is critical for meaning reconstruction. For example, receiving detailed information about the accident is considered important for making sense of the loss. Perceived support may serve as a natural catalyst for adaptation to loss (Shear, 2012).

Perceived justice was also investigated in the context of a mass disaster (Choi & Cho, 2020; Huh et al., 2017). Following the Sewol ferry accident, reactive embitterment was associated with complicated grief, which is likely mediated by the impact of meaning reconstruction. The man-made disaster was reportedly largely preventable, with widespread allegations of negligence and government corruption (Choi & Cho, 2020; Huh et al., 2017). Bereaved parents may have experienced a sense of betrayal that shattered their orienting systems. Feelings of injustice may then have hindered their ability to reconstruct their assumptions of the world and find meaning in the loss (Neimeyer, 2019).

Complicated grief was almost always examined in relation to other psychological and physical conditions (Choi & Cho, 2020; Dyregrov et al., 2003; Feigelman et al., 2011; Field et al., 2014; Huh et al., 2017; Spooren et al., 2001). These studies found that complicated grief was frequently comorbid with PTSD, depression, anxiety, somatic symptoms, insomnia and psychosocial complaints. Parents bereaved by accidents appear to face a high burden of functional impairment. Importantly, complicated grief has been reported to persist even after the treatment of related conditions (Shear et al., 2013). While there are established pathways for the management of all identified comorbidities, there are currently no universal clinical practice guidelines for the treatment of complicated grief (Shear, 2015; Killikelly & Maercker, 2017; Morris et al., 2019).

### Limitations

The methodological rigour of the included literature constitutes a major limitation of this review. Given the context of the research, all of the included studies utilised non-probability sampling techniques, which compromises the generalisability of the findings. Indeed, non-response bias is a major concern in most bereavement research (Lundorff et al., 2017). Many of the included studies also used unvalidated measures for secondary outcomes, which pose a threat to internal validity (Choi & Cho, 2020; Dyregrov et al., 2003; Feigelman et al., 2011; Spooren et al., 2001). Additionally, the included literature is limited by the predominance of cross-sectional designs. When considering the chronicity of complicated grief, longitudinal designs should be considered a research priority (Shear, 2012; Wilson et al., 2022).

### Research and Clinical Implications

There is a paucity of research exploring complicated grief in parents bereaved following accidents. The current literature has focused on the measurement of complicated grief and its associated risk factors. Quantitative research has thus far dominated the field, with a number of instruments now available for the assessment of grief reactions (Wilson et al., 2022). This has allowed for the identification of particularly vulnerable groups of bereaved individuals. Qualitative research has the potential to add depth to the assessment of grief reactions, with a focus on illuminating the lived experiences of those left behind. Future research should consider methodological pluralism as the way forward (Stroebe et al., 2003). While quantitative evaluation is important for diagnostic purposes, it is only through qualitative inquiries that parental grief trajectories can be truly understood.

As the nuances of parental bereavement outcomes from traumatic loss become clearer, it will also be necessary to build upon the current literature by distinguishing between types of accidental death. The included studies focused on either a single form of accident or undifferentiated accidents. This has provided a foundation upon which to explore complicated grief following the accidental death of a child; however, it remains difficult to ascertain potential variability in outcomes across different types of accidents. Future research should further investigate the ways in which specific forms of accidental death impact parental grief symptomology.

In light of the current findings, a number of recommendations are proposed for the treatment of complicated grief in bereaved parents. As a vulnerable population, bereaved parents should undergo routine assessment for symptoms of complicated grief. This is especially important in cases of increased risk, such as sudden or violent death. Practitioners should empower bereaved parents to retain continuing bonds with their deceased child in a culturally appropriate manner. Practitioners should also hold space for bereaved parents to fully comprehend the loss and its significance, with the aim of fostering hope for a meaningful future. Given the importance of perceived support identified in this review, practitioners should encourage bereaved parents to consider participating in a relevant support group in an effort to cultivate a sense of community and facilitate helpful expressions of grief (see Umphrey & Cacciatore, 2011). Frameworks for intervention should always consider the dynamic and highly individualised nature of grief across time.

## Conclusion

Ultimately, there is much still unknown about the convergence of parental bereavement and traumatic bereavement—that is, the sudden or violent loss of a child. It is clear that parents bereaved following accidents are at increased risk for developing complicated grief. Poorer outcomes were identified in relation to the mode of death, relationship type, time post-loss, perceived support, perceived justice and comorbidities. The death of a child is a transformational experience that carries unique challenges for those left behind. It is hoped that the current findings may be used to inform clinical practice guidelines for the treatment of complicated grief.
